# An interactive time series image analysis software for dendritic spines

**DOI:** 10.1038/s41598-022-16137-y

**Published:** 2022-07-20

**Authors:** Ali Özgür Argunşah, Ertunç Erdil, Muhammad Usman Ghani, Yazmín Ramiro-Cortés, Anna F. Hobbiss, Theofanis Karayannis, Müjdat Çetin, Inbal Israely, Devrim Ünay

**Affiliations:** 1 Champalimaud Research, Champalimaud Centre for the Unknown, Lisbon, 1400-038 Portugal; 2 Laboratory of Neural Circuit Assembly, Brain Research Institute (HiFo), University of Zürich, Zürich, Switzerland; 3 UZH/ETH Zürich, Neuroscience Center Zurich (ZNZ), Zürich, Switzerland; 4 ETH Zürich, Computer Vision Laboratory, Zürich, Switzerland; 5 Department of Electrical and Computer Engineering, Boston University, Boston, 02215 MA USA; 6 Departamento de Neurodesarrollo y Fisiología, Instituto de Fisiología Celular, Universidad Nacional Autónoma de México, Mexico City, C.P. 04510 Mexico; 7 Department of Electrical and Computer Engineering, Goergen Institute for Data Science, University of Rochester, Rochester, 14627 NY USA; 8 Department of Pathology and Cell Biology, Columbia University, New York, 10032 NY USA; 9 Department of Biomedical Engineering, İzmir University of Economics, İzmir, Turkey; 10 Department of Electrical and Electronics Engineering, İzmir Democracy University, İzmir, Turkey

**Keywords:** Neuroscience, Biomedical engineering

## Abstract

Live fluorescence imaging has demonstrated the dynamic nature of dendritic spines, with changes in shape occurring both during development and in response to activity. The structure of a dendritic spine correlates with its functional efficacy. Learning and memory studies have shown that a great deal of the information stored by a neuron is contained in the synapses. High precision tracking of synaptic structures can give hints about the dynamic nature of memory and help us understand how memories evolve both in biological and artificial neural networks. Experiments that aim to investigate the dynamics behind the structural changes of dendritic spines require the collection and analysis of large time-series datasets. In this paper, we present an open-source software called SpineS for automatic longitudinal structural analysis of dendritic spines with additional features for manual intervention to ensure optimal analysis. We have tested the algorithm on in-vitro, in-vivo, and simulated datasets to demonstrate its performance in a wide range of possible experimental scenarios.

## Introduction

The efficacy of excitatory synapses changes with development^1^, activity^2^, and learning^3^, and correlates with structural changes of dendritic spines^4–9^. Changes in efficacy and structure can impact subsequent information transmission between inputs across the dendritic arbor^10–12^. Understanding how such changes are physically maintained in the neuron is key to elucidating the mechanisms by which information is stored in the brain. Activity-dependent structural changes at spines can last from minutes to days and are experimentally visualized through multi-time point sampling of z-stack images, often collected over many hours. For example, in an experiment that addresses structural plasticity mechanisms along a dendritic branch using fluorescence imaging, depending on the image acquisition conditions and the type of the neuron that is being imaged, hundreds of spines can be assessed. In a longitudinal study, the total number of spines to be analyzed can reach up to thousands, and analyzing such a dataset manually is tedious and time-consuming.

The focus of the investigation into structural changes of dendritic spine features has thus far been centered on changes in spine head volume, spine neck length, and density of spines at a dendritic segment. Two methods have emerged in the field for the estimation of spine volume, namely integrated fluorescence intensity (IFI) and full-width at half-maximum (FWHM)^8,13^. The IFI method sums all the fluorescence values within a region of interest (ROI) drawn around the spine of interest^7,14,15^. In the FWHM method, an intensity profile over a line perpendicular to the spine neck and passing through the spine head center is used to fit a Gaussian. The half-maximum value of the estimated Gaussian fit is used to estimate the diameter of a hypothetical sphere representing the spine head. IFI is sensitive to fluctuations of fluorescence intensity caused by the imaging system, which can be dramatic; whereas FWHM suffers from over or underestimation if the spine head is not a perfect sphere, as is often the case^16^. In order to overcome any intensity variation due to changes in the expression of fluorescent proteins that might occur during imaging, the IFI volume estimation can be corrected by normalizing by the fluorescence intensity at the nearby dendritic segment after background subtraction^15,17^. As well as the spine head, spine necks have important physical consequences for spine efficacy due to their role in coupling the spine head with the dendritic shaft^18^, and neck features such as width and length can undergo structural modifications that correlate with activity. Variability and fluctuations of total spine length have been used to quantify spine temporal dynamics in the context of population spine motility during development^19–24^ or after plastic changes of the brain network^25^. Additionally, it has been shown that the spine neck gets shorter and thicker as the spine head gets bigger following long-term potentiation (LTP)^9,26^.

In this paper, we present an open-source Matlab-based software called SpineS that allows: (1) Automatic registration of dendritic arbor images collected at consecutive time points to correct for spatial displacements occurring during image acquisition, (2) Automatic detection and segmentation of dendrites and spine heads, (3) Calculation of spine head volumes using IFI and spine neck lengths from two-photon laser scanning microscopy (2PLSM) and confocal laser scanning microscopy (CLSM) image stacks. SpineS also provides tools for reviewing and correcting spine detection errors, dendrite and spine head segmentation, and spine neck paths, as well as a tool to manually estimate FWHM-based spine head volumes. We chose Matlab since it is widely used by biologists for image analysis tasks. The Matlab toolboxes that are necessary to run SpineS are Curve fitting, Image processing, Signal processing, Deep Learning, and Statistics. Here we explain both the front- (graphical user interface, or GUI) and back-end (algorithms) inner workings of SpineS and provide extensive performance analysis on multiple datasets collected in different laboratories using various imaging systems.

Various open-source software packages have been developed for dendritic spine analysis during the last decades, most of which are either no longer available/maintained or are not designed to analyze longitudinal datasets. Neuron-IQ^27^ was a Matlab-based software package that is no longer downloadable. NeuronStudio^28^ was incorporated into the commercial software Neurolucida^29^, and thus requires payment to use. SpineJ^30^ is a recently released good alternative for super-resolution microscopy image stacks, but whether it would work for CLSM or 2PLSM images is unclear. Furthermore, it does not offer time-series analysis. 2dSpAn^31^ and its 3D version 3dSpAn^32,33^ are two currently available software packages for morphological analysis of dendritic spines. Although these software packages do provide good results, they neither offer longitudinal analysis nor have manual correction capabilities. However, an advantage of these tools is that they do not require Matlab to run as our software does. Another Matlab-based tool, Braintown^34^, offers a machine learning-based solution for the automatic detection of spines from two-photon image stacks. Although it is highly accurate at detecting spines, it does not provide further analysis blocks. Apart from these tools, several algorithms have been proposed to quantify various dendritic features over the years. We present a brief non-exhaustive list of dendritic spine analysis literature in Supplementary Table 1.

## SpineS GUI

**Figure 1:**
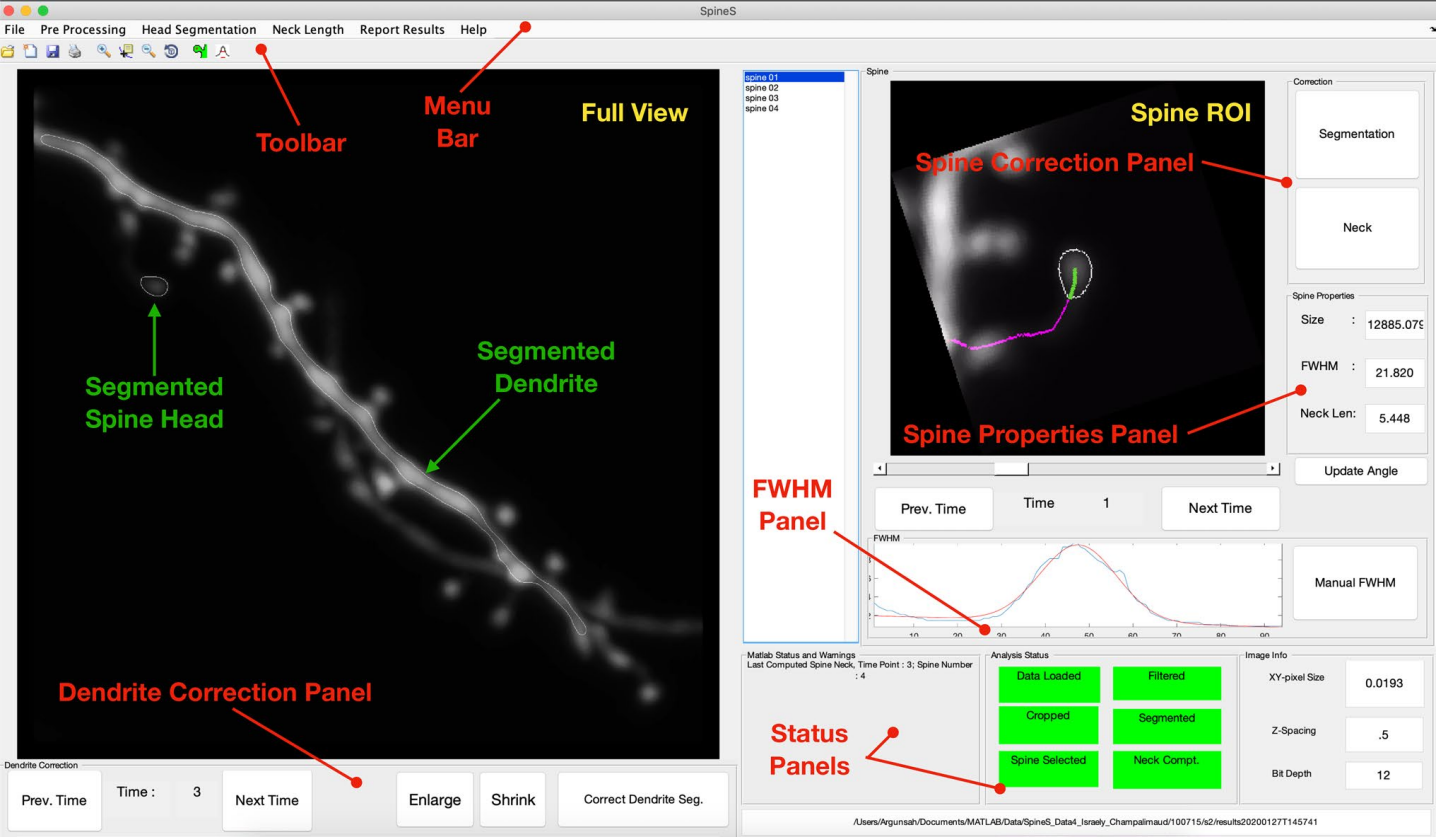
The SpineS GUI allows automatic analysis of dendritic spines in combination with interactive manual correction tools. The *Full View* pane shows a maximum intensity projection (MIP) of the first time point of the dendrite to be analyzed after loading the data. SpineS automatically detects various dendritic features, and segments dendrites and dendritic spines for all time points. Detected feature points and segmentations are used to quantify the number of spines, spine volumes, and spine neck lengths. There are several tools for users to correct mistakes made by the program.

The SpineS toolbox has multiple individual functions that can be called independently to perform different steps of spine analysis. Additionally, we have developed a GUI that includes all the necessary components to perform a complete analysis (Fig. 1 and (Supplementary Fig. S1)). In this section, we introduce the components of the SpineS GUI and the necessary instructions to conduct a full analysis.

### Steps to analyze a dataset

The first step that is carried out by the SpineS software is loading the data (Supplementary Fig. S2A). Since different laboratories use different imaging systems and image formats, data specifications can be very different. In SpineS, we use the bio-formats library provided by the open microscopy environment project to support all microscopy platforms^35,36^. The software computes the maximum intensity projection (MIP) of the first time point (first z-stack) to display the image to the user (see Fig.  1). Upon loading the data, the software first registers consecutive time points to correct movement artifacts and then detects spine head centers, dendrite boundaries, and the points in which spines and dendrites connect (neck base). Once this automatic detection is complete, users can add missing spines manually (Supplementary Fig. S2B). The next step is the segmentation of spine heads and dendrite boundaries. Following automatic dendrite segmentation, the *Dendrite Correction Panel* shown in Fig. 1 allows the user to fine-tune the segmentation. Whilst editing the segmentation, it is possible to enlarge or shrink the entire segmented dendrite. The user can shrink or expand the boundaries using an active contour-based segmentation correction algorithm^37^. To allow for correction at a finer scale, the *Correct Dendrite Seg* button allows the user to correct specific parts of the segmentation mask (see Supplementary Fig. S3).

Once the automatic spine head segmentation process is completed, the user can navigate through the time points to inspect the quality of the segmentation and refine them if necessary by using the *Segmentation* button in the *Correction* panel (shown in Fig. 1). Once pressed, this button will present a new image window with the segmentation in a draggable correction mesh (see Supplementary Fig. S4).

After all head segmentation corrections are completed, the automatic spine neck path extraction algorithm can be run (Supplementary Fig. 2D). Erroneous neck paths can be corrected using the *Neck* button in the *Correction* panel shown in Fig. 1 by providing a new base point on the dendrite.

If FWHM-based volume estimation is desired, the spine orientation should be changed by using the slider below the *Spine ROI* panel such that the spine head is oriented vertically as shown in Fig. 1-Spine ROI. When orientation is modified at an earlier time point, all consecutive time points for that spine are registered automatically. FWHM requires the selection of a rectangular region including the spine head center and surrounding background. This process can be started with the FWHM icon (Supplementary Fig. 2B).

Spine properties such as IFI-based head volume, FWHM-based head volume, and neck length are shown in the spine properties panel (see Fig. 1) after the completion of the necessary steps. Throughout the analysis, progress can be followed in the *Analysis Status* panel as the background color of each process turns from red to green upon completion (see Fig. 1). Analysis results such as the number of detected spines over time, normalized and non-normalized spine head volumes, and spine neck lengths can be exported to MS Excel (Supplementary Fig. 2E). Additionally, plots will be created for each reported result and the region of interest (ROI) for each detected spine, as well as segmentation masks, will be saved in the results folder upon the completion of the analysis.

## Methods

**Figure 2:**
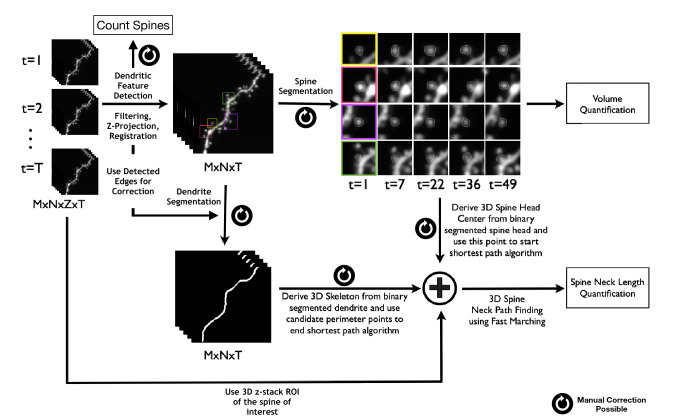
Workflow of SpineS. Z-stacks (M $\times$ N $\times$ Z) from multiple time points (T) are analyzed. Each z-stack is imported and registered. MIPs are then computed and filtered using median filtering. Dendritic spine heads are detected using a CNN and segmented using a watershed segmentation algorithm followed by a graph-based clustering. Each spine volume is estimated using the integrated fluorescence intensity (IFI) of the spine head within the segmentation region. The intensities in the region are normalized using the median fluorescence intensity of the dendrite at the corresponding time point after background fluorescence subtraction. Neck paths from the spine head center to the closest geodesic point on the dendrite are computed using the fast-marching algorithm by imposing empirically set constraints.

In this section we present the image processing workflow that we created that constitutes the SpineS tool. The flow diagram of the analysis process can be seen in Fig. 2 and Supplementary Fig. 3, and the details for each section are explained below.

### Registration

During the imaging of dendritic segments, mechanical movements of the sample or the dynamic nature of dendrites and spines can cause movements in the images. In order to correct for such translational movements, we apply a two-step registration procedure.

#### Global registration

Global registration aligns two consecutive image stacks at sub-pixel precision using a discrete Fourier transform (DFT) based algorithm^38^. This algorithm uses selective upsampling by a matrix-multiply DFT to reduce computation time and memory while preserving accuracy.

#### Local registration

Although the global registration broadly aligns two consecutive time points well, some local misalignments may remain due to orientation and shape changes of dendritic spines. As a result, the spine of interest might slightly shift from the center of the ROI. In SpineS, we force the spine of interest to stay at the center of the ROI using local registration to avoid analyzing an incorrect spine that might appear in the ROI after the global registration. We achieve this using a Normalized Mutual Information (NMI)-based local registration method^39^. In this method, after global registration, we shift the ROI within a small neighborhood and measure the NMI with the ROI in the previous time point. Finally, we take the ROI with the highest NMI and use it in the analysis.

### Automatic spine detection and classification

**Figure 3:**
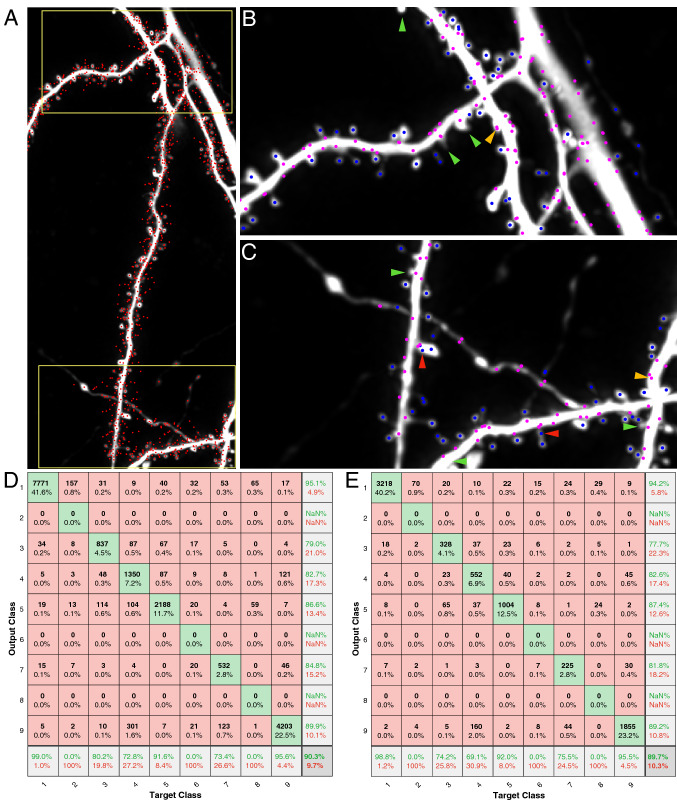
Automatic detection of various dendritic features via SURF features and CNN-based classification. (**A**) Candidate SURF locations are marked with red dots. $3.395\times 3.395\,\upmu {\mathrm{m}^2}$ ROIs around each SURF location are fed into the dendritic feature classification network (CNN). (**B,C**) The network has 9 output classes. Here we show two of the classes: Dendritic spines in blue and dendrite edges in magenta. Green arrows show undetected spines. Red arrows are misdetections and yellow arrows are misclassifications. (**D**) Confusion matrix for the training dataset. (**E**) Confusion matrix of the test dataset. Classes are 1: spines, 2: multiple overlapping spines, 3: spine neck base, 4: dendrite edge, 5: dendrite, 6: spine neck, 7: spine head edge, 8: bouton/axon (some datasets contained these structures as well), 9: noise.

We developed a method for automatic spine detection and classification using speeded-up robust features (SURF)^40^ and convolutional neural networks (CNN) (Supplementary Fig. S5) inspired by earlier works in which neural networks were used to detect dendritic spines^34^, and CNN-based detection was applied to ROIs previously extracted by a feature detector^41^. SURF is a well-known method to automatically extract distinctive features from an image. We observed that, when applied to an image of a dendritic branch, the features identified include most of the spine centers as well as many other locations, as shown in Fig. 3. We cropped a $97\times 97$ sized ROI (corresponding to $3.4\,\upmu {m}\times 3.4\,\upmu {m}$) centered around each SURF feature location. When determining this ROI size, we searched the range [$1\,\upmu {m}$, $4.8\,\upmu {m}$] with increments of  $1.4\,\upmu {m}$ and selected the ROI size which gave the highest validation accuracy. We manually assigned each ROI to one of nine class labels based on the location of the SURF features. The labels and some samples from each class are shown in Supplementary Fig. S6. We extracted 26705 ROIs in total from 97 dendritic branch images obtained from 20 datasets. The number of ROIs from each class is as follows: Class 1: 11106 spine heads (3627 of them from previously published open data^42^), Class 2: 268 multiple spines, Class 3: 1485 spine-dendrite edges, Class 4: 2654 dendrite edges, Class 5: 3480 dendrites, Class 6: 165 spine necks, Class 7: 1023 spine head sides, Class 8: 184 axons/boutons, and Class 9: 6340 noisy regions that do not contain spines or any other dendritic features but reflect natural noise (random variation of brightness) observed in the images. We used 70$\%$ of the ROIs during training and the remaining 30$\%$ as a validation set to create the confusion matrices presented in Fig. 3D,E. For in-vivo detection experiments, we pooled all non-spine classes (Classes 2 to 9) to make one big non-spine class and trained the network with only two classes. Due to the Signal-to-Noise Ratio (SNR) and resolution differences between in-vivo and in-vitro conditions, the CNN with nine classes did not perform as accurately as the CNN with two classes. We trained a CNN using the training set and selected the model with the best validation set accuracy as the final model. Our CNN architecture, depicted in Supplementary Fig. S5, consists of six blocks each containing a convolutional layer followed by batch normalization, swish activation, and max pooling. The convolutional blocks are followed by three fully connected layers with sizes 4096, 256, and 9.

### Dendrite segmentation

Dendrite segmentation starts by filtering the MIP image using a 2D-median filter and binarizing via Otsu thresholding^43^ to get a rough segmentation of the dendritic branch including spines. The medial axis of the dendrite is computed by applying a fast marching distance transform^44^ on the dendritic segment between the two ends of the dendrite. A locally adaptive-sized disk-shaped structuring element around the medial axis of the dendrite is applied to remove the connected spines. Additional refinement is performed by assuming that the dendrite diameter remains constant within the local field of view.

### Spine head segmentation

We use a multilevel algorithm for spine head segmentation^45^. The initial segmentation in the pipeline is performed using a watershed algorithm^46^. Watershed segmentation requires a seed point to start and a boundary to determine where to stop the segmentation. When determining the seed points, we assume that the spine head contains a maxima region which we extract using a morphological transformation^47^. In addition to the maxima regions, we use automatically detected spine head centers to capture all dendritic spines in the field of view. We then feed these regions as seeds to the watershed to start the segmentation. We determine the boundaries of the segmentation using Otsu’s thresholding^43^. If some spines are below the threshold, we add circular mock boundaries around the detected spine centers. Watershed segmentation usually finds larger boundaries than manual segmentation by experts. We, therefore, incorporate a second level of the segmentation algorithm, which takes each previously found component in the region of interest and refines it using a modified version of a graph-theoretic algorithm for arbitrary shape detection^48^. This algorithm performs segmentation inside the region found by watershed segmentation to generate multiple clusters. These clusters are then combined using hierarchical clustering to obtain ten clusters, which are formed as quasi-concentric connected components. We exclude the outermost clusters and use the remaining ones to specify the final segmentation.

### Finding the spine neck path

Neck length computation is a challenging task due to spine shape variations and neck motility. We begin computing the neck length by eroding each slice of the dendritic branch image with a disk-shaped structuring element to reduce spurious paths. Then, a multi-stencil fast marching (MSFM) method^44,49^ is applied to compute the 3D distance map using the spine head center as the source point. The Runge-Kutta algorithm^50^ is applied on the 3D distance map to compute the shortest paths (geodesic) from *N* points on the dendrite perimeter to the spine head center. These *N* points are selected by finding the *N* nearest points from the spine head center to the dendrite perimeter using the Euclidean distance as a metric.

The crucial final step is the selection of the correct neck path. A simple approach would be to select the path with the minimum length (Eq. 1), but that would be suboptimal due to the motile and sometimes non-linear nature of spine necks. Therefore, using the mere path length as a constraint is not enough. We introduced two additional constraints to select the path with the best geodesic approximation: Path complexity (Eq. 2) (L1-norm of path derivatives), and path smoothness (Eq. 3) (L1-norm of image intensities along the path). We select the neck path that collectively has the lowest value for these three constraints (Eq. 4).

1 $$\begin{aligned}&L_P=\int _P{dS} \end{aligned}$$

2 $$\begin{aligned}&C_P=\left\| {\dfrac{\delta {P}}{\delta {x}}} \right\| _1 + \left\| {\dfrac{\delta {P}}{\delta {y}}} \right\| _1 + \left\| {\dfrac{\delta {P}}{\delta {z}}} \right\| _1 \end{aligned}$$

3 $$\begin{aligned}&S_P=\left\| {\dfrac{dV(x_p,y_p,z_p)}{dI}} \right\| _1 \end{aligned}$$

4 $$NeckPath = \mathop {{\rm{argmin}}}\limits_{{\rm{P}}} \left(\frac{{L_{p} }}{{max(L_{p} )}} + \frac{{C_{p} }}{{max(C_{p} )}} + \frac{{S_{p} }}{{max(S_{p} )}}\right)$$

To compute the neck path, we remove the portion of the path that resides inside the segmented spine head and dendrite borders.

**Figure 4:**
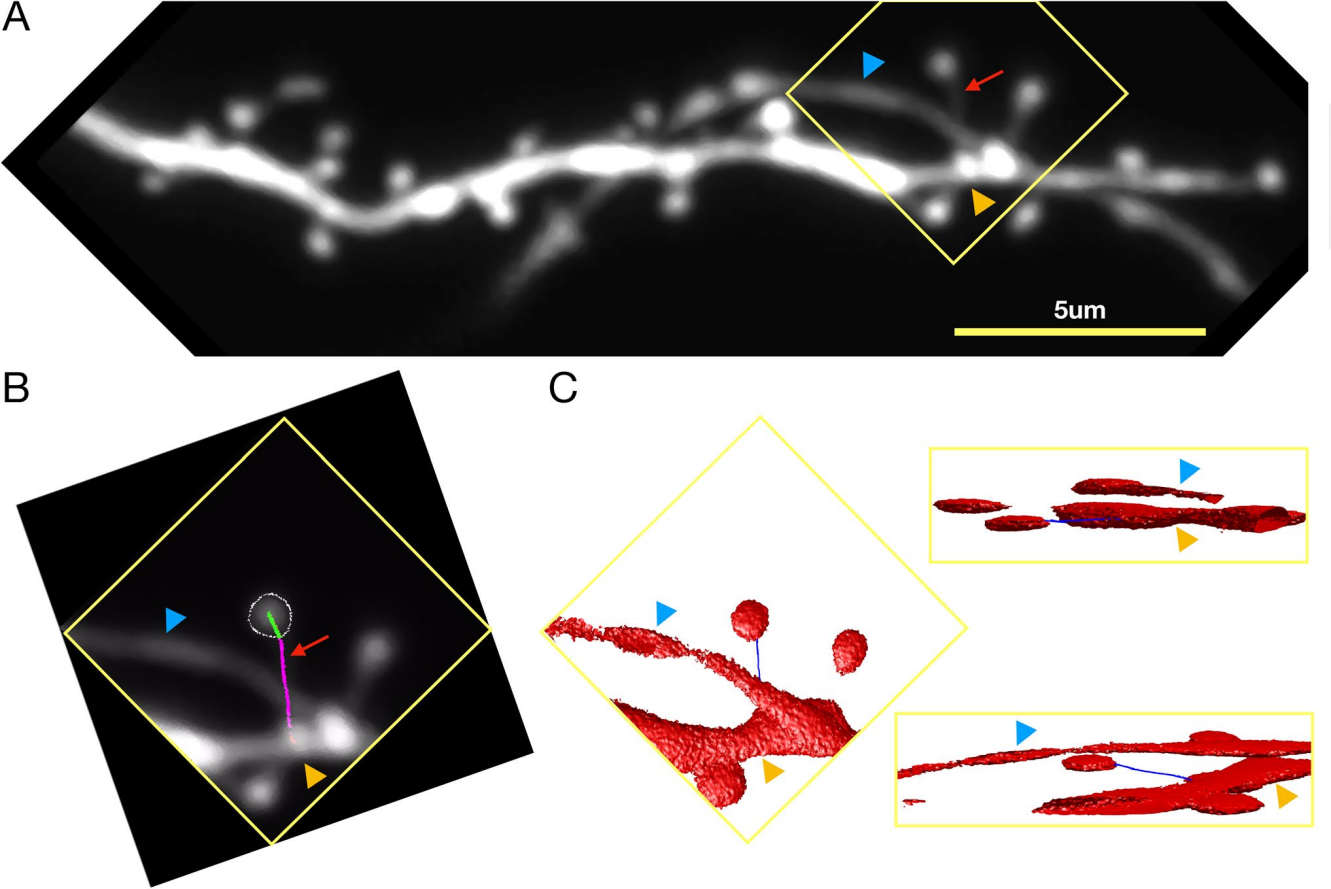
Finding spine neck path in 3D. (**A**) Two-photon microscopy image of a dendrite of a hippocampal CA1 pyramidal neuron in-vitro. The blue arrow indicates the nearby process. The orange arrow indicates the parent dendrite. The red arrow indicates the spine neck. Image contrast is increased to improve the visibility of the spine neck. (**B**) ROI in yellow square in **(A)**. The path from the parent dendrite to the spine head border is shown in magenta. The path from the spine head border to the spine head center is shown in green. (**C**) 3D rendered version of B showing the neck path from three different orientations, showing that the spine neck is correctly traced from the spine center to the dendrite but not to a nearby process.

An example spine neck path is shown in Fig. 4B. The green line shows the computed path inside the spine head, hence not used to calculate the neck length. The magenta part shows the remaining neck path. Figure 4C shows that the spine base point is attached to the dendrite (green arrowhead) but not to a nearby passing axon (blue arrowhead).

### Volume estimation

#### Integrated fluorescence intensity

Intensity values within the segmented spine head are summed and the background fluorescence intensity (the minimum intensity value in the spine field of view (FOV) $\times$ area of the segmented spine) is subtracted from the summed intensity value. Finally, the intensity is normalized to the dendrite to account for relative changes in fluorescence level over time that might be caused by bleaching, movement, or imaging laser power fluctuations^7,15,17^.

In earlier studies, summed intensities in the spine head were normalized using the highest intensity value of the dendrite portion closest to the spine. However, this approach is problematic for two reasons. Firstly, if the collected images are saturated, the maximum recorded value is not a true representation of the brightness, thus maximum intensity normalization produces misleading results. Secondly, since MIP images are collapsed along the z-axis, spines on this axis often appear as bright hotspots. If a nearby spine of interest is normalized to these hotspot-like points, one may end up normalizing to a floating reference. We believe this problem is either overlooked or solved but not reported in publications. In order to overcome this issue, we use the median intensity value inside the segmented dendrite to normalize, instead of the maximum intensity value. SpineS also reports the raw values for summed intensities, background fluorescence intensity, and fluorescence intensity distribution within the segmented dendrite so that users can select the most relevant metric for their study.

#### FWHM-based volume estimation

It is often assumed that a spine has a spherical shape^5,8,51^. The FWHM method is used to find the radius, *r*, of a sphere that encloses the spine. Then, the spine volume is computed using the formula $4/3\pi {r^3}$.

The fluorescence intensity profile is measured along a line parallel to the postsynaptic density (PSD), the active zone of the synapse normally located on the opposite side to the spine neck. A Gaussian curve is fitted to this intensity profile. Finally, the FWHM value of the Gaussian curve becomes the diameter (*R*) of the sphere. A benefit of using an FWHM method is that it returns a volume in real units (as opposed to arbitrary units for IFI) and is invariant to changes in fluorescence intensity. However, there are some weaknesses of FWHM-based volume estimation. This approach produces accurate results if the spine shape is close to spherical. However, dendritic spines are mostly non-spherical. Moreover, small variations in the rotation angle, which is normally set manually, may change the estimated spine volume significantly. Since it is often not trivial to determine where the PSD is in the MIP image, a crucial step to draw the fluorescence intensity profile, the current version of SpineS does not have a feature for finding the best orientation automatically.

#### Ethics declarations

Four different datasets from three different institutions were analyzed in this study. Experiments in Dataset $\#$1 were carried out in accordance with European Union regulations on animal care and use and with the approval of the Champalimaud Centre for the Unknown Ethics Committee and the Portuguese Veterinary Authority (DGAV). Experiments in Dataset $\#$2 were reviewed and approved by the ethical committee from the Instituto de Fisiología Celular de la Universidad Nacional Autónoma de México in accordance with the guidelines on the use of animals from the Society for Neuroscience. Experiments in $\#$3 and $\#$4 were performed according to the guidelines established by the European Union regulations and were previously approved by the institutional animal welfare body (ORBEA) and the Portuguese Veterinary Authority (DGAV). All the animal experiments in this study are reported in accordance with ARRIVE guidelines (https://arriveguidelines.org). Dataset$\#$5 experiments were performed by the animal welfare guidelines of Tsinghua University.

## Results

SpineS has three major analysis modules: detection, spine head segmentation, and spine neck length quantification. We presented the performance of the detection module in Fig. 3D,E. Below we present quantitative and qualitative performance comparisons for segmentation and neck length quantification. Since there is no benchmark dataset for dendritic spine analysis, we have presented quantitative comparisons when possible, and used qualitative measures otherwise (e.g. spine heads have been shown to shrink after the induction of Long Term Depression (LTD). If our analysis goes hand in hand with the expected phenomenology, we accept that as a good quality result).

### Fluorescence intensity invariant performance in spine segmentation and volume estimation

**Figure 5:**
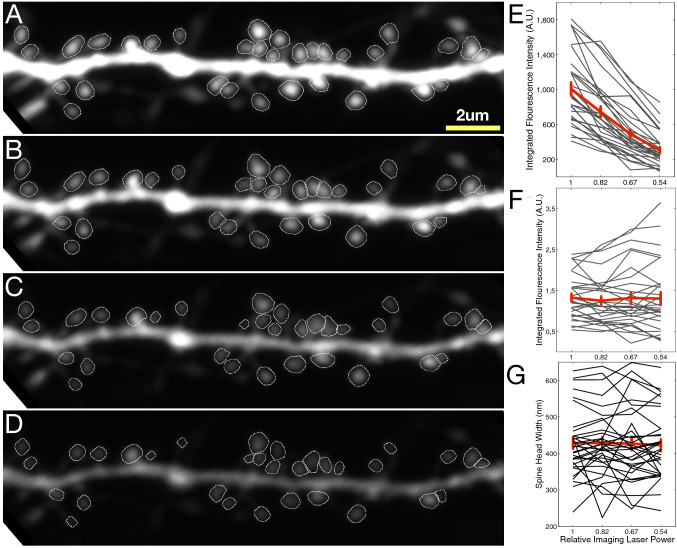
Performance of segmentation and volume estimation are not intensity dependent. (**A–D**) Maximum intensity projection of two-photon microscopy images collected using different imaging laser powers as the tissue was perfused with ACSF containing TTX to prevent short-term plastic changes at the dendritic branch. (**E**) Individual (gray) and average (red) intensity-based volume estimation of segmented spines. (**F**) Normalised integrated fluorescence intensity after normalisation with median intensity value at the dendrite. (**G**) FWHM estimated spine head widths.

Since our IFI-based volume estimation process depends on the quality of the spine head segmentations, we checked if significant fluorescence changes affect the segmentation performance, as well as verifying if changes in fluorescence intensity affect eventual volume estimation. We collected a dataset by repeatedly imaging the same dendritic segment in-vitro using different laser intensities. To ensure that no significant biological remodeling occurred either at the dendrite or the spines over the course of the experiment, we added tetrodotoxin (TTX) into the artificial cerebrospinal fluid (ACSF) used to perfuse the neuron during the imaging session. TTX is a sodium channel blocker that acutely blocks action potential firing, and TTX application changes spine volumes only upon prolonged application^52^. We analyzed the images to to investigate if spine segmentation performance and resulting IFI volumes were sensitive to different levels of fluorescence. Fig. 5A–D shows MIP images and segmented spines of the same dendritic segment imaged using four different imaging laser powers. The segmentation of spines was not affected by changes in fluorescence intensity. The average IFI drops as the imaging laser power decreases (see Fig. 5E); however, normalization to the dendrite recovers the expected volume invariance of the spines (see Fig. 5F). As mentioned earlier, spine head width which is estimated using FWHM is not affected by intensity fluctuations, even without normalisation (see Fig. 5G).

### Comparison with manual/expert annotations

**Figure 6:**
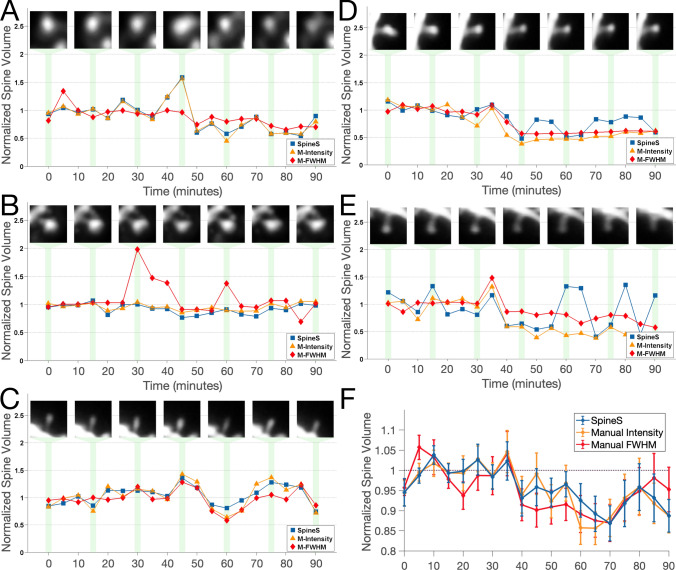
Comparison of automatic segmentation with manual segmentation and manual FWHM based volume estimation methods. (**A–E**) Five examples from a chemical LTD experiment. SpineS: IFI based volume using automatic segmentations; manual Intensity: IFI based volume using manual segmentations by an expert; manual FWHM: FWHM based volume quantified by a different expert. The chemical agent DHPG is applied before the 30 min time point. Volumes are normalised to the average baseline volume. Similarity scores between the different analysis methods are as follows: (**A**) $SS_{S-MI}=97.09$, $SS_{S-MF}=83.85$, $SS_{MI-MF}=84.23$, (**B**) $SS_{S-MI}=94.34$, $SS_{S-MF}=78.81$, $SS_{MI-MF}=81.16$, (**C**) $SS_{S-MI}=91.87$, $SS_{S-MF}=87.78$, $SS_{MI-MF}=88.16$, (**D**) $SS_{S-MI}=83.85$, $SS_{S-MF}=87.35$, $SS_{MI-MF}=89.86$, (**E**) $SS_{S-MI}=72.66$, $SS_{S-MF}=69.78$, $SS_{MI-MF}=77.61$, (**F**) Average of 27 spines. Comparisons for all 27 spines can be found in Supplementary Table 2.

We compared the volumes of 27 spines from nine different dendritic segments collected for 90 min at 5-min intervals with human expert analysis results (Fig. 6). It has been shown that application of the mGluR1-5 agonist (S)-3,5-Dihydroxyphenylglycine (DHPG) induces long-term depression (LTD) at dendritic spines^53^. We analyzed this dataset using SpineS and compared the results of the software with those obtained by two experts in this domain, each manually performing one of the two spine volume estimation methods explained earlier—manual segmentation for IFI estimation and manual spine alignment for FMWH estimation with a tool designed for ImageJ. Spine volumes for all methods were normalized to the baseline (pre-DHPG application) values. To compare methods we used the symmetric mean absolute percentage error (*sMAPE*)-based similarity score (SS). The *sMAPE* is a robust measure for trend comparisons in time series data:

5 $$\begin{aligned} sMAPE^{spine} = 100\dfrac{1}{t}\sum _{t=1}^{t}\dfrac{\Vert SpineS_i-Expert_i\Vert _1}{\Vert SpineS_i+Expert_i\Vert _1} \end{aligned}$$

where, *t* represents time, $SpineS_i$ and $Expert_i$ represents the volume estimations of SpineS and Expert at time point *i*, respectively.

Using *sMAPE*, we compute the similarity score as follows:

6 $$\begin{aligned} SS^{spine} = 100(1-sMAPE^{spine}). \end{aligned}$$

Comparisons of normalized volume results derived from SpineS segmentation, manual segmentation and manual FWHM estimation are given in Supplementary Table 2. Similarity scores of IFI-based volume estimates from manually segmented spine heads and SpineS outputs suggest that automatic segmentation yields very similar ($\mu$ = 90.28%; $\sigma$ = 5.83%) spine head segmentations to the experts’ since both use an intensity-based volume estimation method. We also compared our results with the manual FWHM volume estimation results to see how comparable these two common volume estimation methods are. Figure 6A–E shows five representative examples of analysis results. The caption contains the similarity scores of pairs of methods for a given spine. In Fig. 6F we see that on average, all three methods show statistically similar outputs (all pairwise t-test $p>0.5$).

Despite the statistically similar results, we believe that the IFI approach used in SpineS is the more robust estimation method. As explained above, the FWHM-based approach might lead to erroneous estimates if the spine head shape diverges from being spherical. The IFI approach gives an arbitrary volume unit, which is sufficient if the focus of the study is relative volume change. If real units of volumes are required, the user should perform a manual FWHM procedure on several spines visually selected for sphericity, to find a conversion factor into $\upmu {\mathrm{m}^3}$ for the arbitrary IFI volume units.

### Performance on structural plasticity data

**Figure 7:**
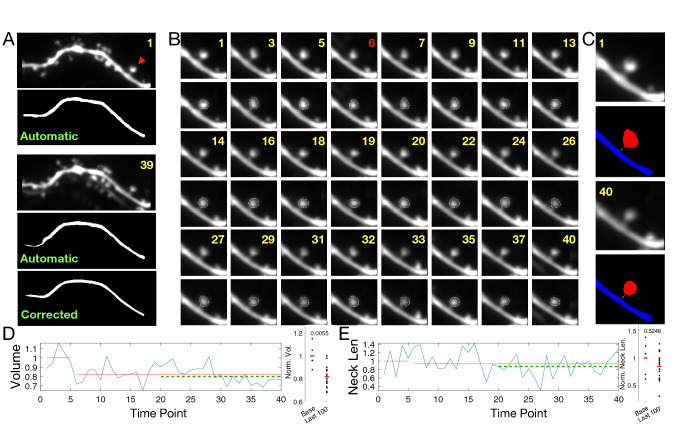
Analysis of glutamate uncaging induced single spine LTD experiment. (**A**) The dendritic segment before (upper panel) and after (lower panel) stimulation. At the first time point, the dendrite segmentation is perfect but at a later time point, fluorescence changes lead to a problematic segmentation which is later corrected by manual interaction. The red arrowhead indicates the stimulated spine. (**B**) Spine head segmentation for 40 consecutive time points. Images were taken once every 5 min. Upper rows—unsegmented image; lower rows—segmented image. Stimulation was performed at the 6th time point. (**C**) Spine before (upper panel) and after (lower panel) undergoing LTD. Median filtered images are above, and segmented images with neck paths are below (Blue: dendrite, Red: spine head, White: spine neck). (**D**) Normalized volume and spine neck results (blue: normalized trends, Red: linear fits for before and after stimulation. Green: average of last 20 time points).

One important application area of any spine analysis software is spine head volume and neck length tracking of single dendritic spines. In order to demonstrate the performance of our software for such experimental tasks, we collected images before and after the induction of LTD at a single spine using glutamate uncaging. Figure 7A shows the dendrite of interest before the stimulation. An LTD-inducing glutamate uncaging stimulation was delivered before the 6th time point. Figure 7B shows the ROI of the stimulated spine over time. Figure 7C,D show the persistent shrinkage of the stimulated spine after stimulation. This form of stimulation does not appear to affect the spine neck length (see Fig. 7D).

Two more plasticity experiments are presented in Supplementary Fig. S7 and Supplementary Fig. S8. In S7 LTD is induced chemically, thus affecting all the dendritic spines, which would be expected to shrink. 53 out of 57 spines analyzed indeed exhibit this volume shrinkage over time. Supplementary Fig. S8 presents the results of a chemical long term potentiation (LTP) experiment. On average spines get approximately $1.5\times$ bigger after the chemical stimulation which is captured by the SpineS analysis.

### Performance on in-vivo functional calcium imaging data

Although SpineS was not designed to analyze in-vivo and/or functional data, we performed a preliminary analysis on a recently released public dataset^54^. Despite the fact that our CNN was not trained using any in-vivo datasets, the algorithm can find many dendritic spines robustly, as long as the imaging power and SNR are high (Supplementary Fig. S9). We first tested our initial 9-class CNN classifier for the detection but performance was low (data not shown). To improve this, we grouped all the non-spine classes to create one big non-spine category and trained the same CNN again but this time only using two classes.

### Performance on simulated structural data

**Figure 8:**
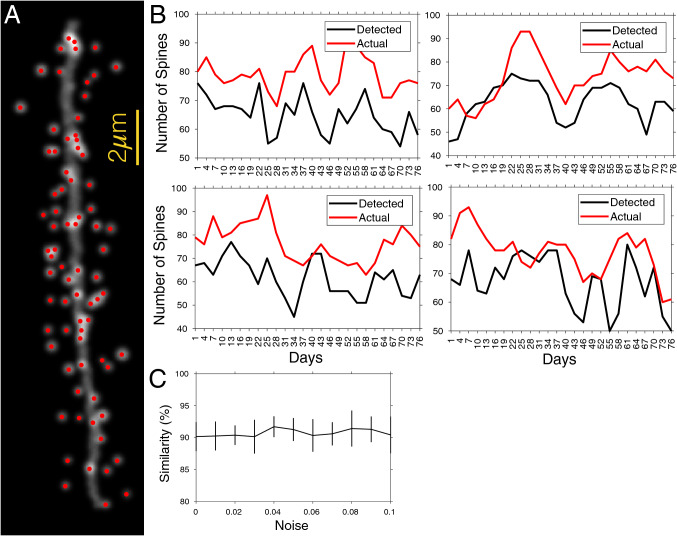
Dendritic spine gain/loss analysis using spine detection on simulated data. (**A**) An example simulated dendritic branch and with detected spines. (**B**) Actual and detected number of spines in four different examples of a simulated dendritic branch over 75 days with realistic spine turnover dynamics. (**C**) The similarity of temporal patterns between actual and detected spine numbers under different simulation noise conditions. Graphs show mean ± std. The similarity score is calculated using sMAPE-based similarity score quantification (mean ± std).

Finally, to test the performance of SpineS for spine counting over time, we applied SpineS to simulated dendrites using a simulation code^55^ that creates dendritic segments and spines that evolve over time according to parameters estimated from in-vivo two-photon imaging experiments. Simulated dendritic branches look very realistic (Fig. 8A). We ran the simulation 100 times. Each simulation created 26 versions of the same dendrite that evolved over 75 days. Analysis of spine number on individual dendrites over time (Fig. 8B) and the average of all 100 simulations under different noise conditions (Fig. 8C) show a high correlation between the actual spine count and detected spine count.

## Datasets

**Table 1:** Details of the datasets used in the study.

Data$\#$	Modality	In-	Obj.	NA	FOV (μm^2^)	Voxel size (μm^3^)	Bit depth
1	2PLSM	Vitro	$60\times$	0.9 air	$19.8\times 19.8$ $\upmu {\mathrm{m}^2}$	$0.0194\times 0.0194\times 0.5$	12
2	2PLSM	Vitro	$63\times$	1.0 water	$20.1\times 20.1$ μm^2^	$0.0196\times 0.0196\times 0.5$	12
3	CLSM	Vitro	$40\times$	1.4 oil	$67.6\times 67.6$ μm^2^	$0.066\times 0.066\times 0.23$	16
4	CLSM	Vitro	$63\times$	1.4 oil	$31.4\times 31.4$ μm^2^	$0.0306\times 0.0306\times 0.41$	8
5	2PLSM	Vivo	$25\times$	1.05 water	$158.7\times 158.7$ μm^2^	$0.155\times 0.155$	16
6	2PLSM	Simulated	–	–	$36.9\times 36.9$ μm^2^	$0.072\times 0.072$	8

The last column presents the median filter window size we used during the analysis that can be used as a guideline by the users.

We tested our software on multiple datasets acquired in different laboratories. Datasets 1–4 are used to show the performance of the entire workflow on different types of in-vitro data. Dataset 5 is from an in-vivo calcium imaging experiment. Surprisingly, despite the data being in-vivo and functional (calcium imaging) rather than structural (no structural labeling), and the fact that our network does not include any in-vivo training data, dendritic spines were correctly detected for the high signal-to-noise ratio dataset (here we present only qualitative results since the data does not have manual annotations). Dataset 6 is a simulated dataset acquired using a simulation algorithm that was used previously to analyze dendritic spine turn-over rates for hippocampal and cortical spines^55^. Since the algorithm was designed to simulate two-photon imaging of dendritic segments, we wanted to see if we could detect dendritic spine gain and loss over time using our detector. Figures 4, 5, and 6 were produced using Dataset 1,and Figure 7 using Dataset 2. Supplementary Figures 3, 5, and 6 were the analysis results of Datasets 3 and 4, respectively. The acquisition summary of these datasets is shown in Table 1 and the details are explained below.

### Dataset 1: in-vitro two-photon structural imaging

This dataset comprises images collected during glutamate uncaging-based single spine plasticity experiments. Hippocampal neurons from mouse organotypic slice cultures postnatal days 7–9 were transfected using biolistic gene transfer with gold beads (10 mg, 1.6 μm diameter, Biorad) coated with Dendra-2 (Evrogen) plasmid DNA (100$\mu$g) or AFP using a Biorad Helios gene gun after 6 or 7 days in-vitro (DIV). Imaging experiments were performed 2 to 5 days post-transfection. Slices were perfused with artificial cerebrospinal fluid (ACSF) containing 127 mM *NaCl*, 2.5 mM *KCl*, 25 mM $NaHCO_3$, 1.25 mM $NaH_2PO_4$, 25 mM D-glucose, 2 mM $CaCl_2$, and 1 mM $MgCl_2$ (equilibrated with $O_2$ $95\%$, CO2 $5\%$) at room temperature at a rate of 1.5 ml/min. Two-photon imaging and uncaging were performed using a galvanometer-based scanning system (Prairie Technologies, acquired by Bruker) on an Olympus BX61WI equipped with a $60\times$ water immersion objective (0.9 NA), using a Ti:sapphire laser (910nm for imaging Dendra; Coherent) controlled by PrairieView software. Z-stacks ($0.5 \; \upmu {\text{m}}$ axial spacing) from secondary or tertiary dendrites from CA1 neurons were collected every 5 min for up to 4 hours. $1024\times 1024$ pixel, xy pixel size = $0.0193\mu {m}$ , FOV $19.8\times 19.8\mu {m}$.

### Dataset 2: in-vitro two-photon structural imaging

This dataset comprises images collected during glutamate uncaging-based single spine LTD experiments. Cultured hippocampal slices were prepared from postnatal day 7–10 mice SHANK3 (Jax, no. 017889)^56^. The cultures were transfected by Gene gun (Bio-Rad) with AFP after 3–6 days in vitro (DIV). Slices were perfused with ACSF containing 127 mM *NaCl*, 2.5 mM *KCl*, 25 mM $NaHCO_3$, 1.25 mM $NaH_2PO_4$, 25 mM D-glucose, 2 mM $CaCl_2$, and 1 mM $MgCl_2$ (equilibrated with O2 95% $CO_2$ 5%) at room temperature at a rate of 1.5 ml/min. Two-photon imaging was performed using an LSM 710 microscope (Zeiss) based on a galvanometer scanning system controlled by Zen black software, equipped with W Plan Apochromat $63\times$ water immersion objective (1.0 NA). The light source was a Ti:Sapphire laser (Chameleon Ultra II, Coherent) tuned to 910 nm for imaging and 720nm for uncaging experiments controlled by Zen black software. Z-stacks (0.3 μm axial spacing) from secondary or tertiary dendrites from CA1 neurons were collected every 5 min up to 4 h. For uncaging LTD experiments, the laser was positioned at  0.5 μm from the center of the spine, then light pulses were delivered at a low frequency to induce LTD.

### Dataset 3: in-vitro confocal structural imaging

This dataset comprises images collected during chemical LTD induction. Hippocampal organotypic cultures were made from P6/7 Wistar rats and transfected using biolistic gene transfer with gold beads coated with mCherry. Images were acquired every 10 min at DIV9 from a secondary dendrite of a CA1 pyramidal neuron. Confocal image stacks were collected using a $40\times$ objective with $4\times$ zoom. Synaptic depression was induced by bath application of (RS)-3,5-dihydroxypheylglycine (DHPG,  50  μM) for 5 min^57^.

### Dataset 4: in-vitro confocal structural imaging

This dataset comprises images collected during chemical LTP induction. Hippocampal slices from Wistar rats on postnatal day 7 were transfected with CMV-eGFP 4 days before imaging. Imaging was done at day 15 in vitro. Images were acquired at intervals of 5 min. The microscope was a Zeiss LSM 710 AxioObserver, 63$\times$ oil immersion objective (1.4 NA), Emission wavelength 574 nm, excitation 488 nm, EGFP, pixel size $0.0306\times 0.0306\times 0.0408\, \upmu {\mathrm{m}}$, zoom 4.3.

### Dataset 5: in-vivo functional imaging

This dataset has recently been made openly available^54^. Adult mice at 8–16 postnatal weeks were housed under a reverse light cycle. All experiments were carried out using two-photon microscopes on head-fixed, awake mice. All experiments were performed in accordance with the animal welfare guidelines of Tsinghua University. A custom-designed two-photon microscope was used to acquire low-SNR and high-SNR time-series images simultaneously. The maximum FOV was about $720\,\upmu {\mathrm{m}}$. The typical frame rate was 30 Hz for $512\times 512$ pixels, and the volume rate was decreased linearly with the number of planes to be scanned. For functional imaging of neural activity, transgenic mice expressing Cre-dependent GCaMP6f genetically encoded calcium indicator (GECI) were used. Mice were anesthetized with $1.5\%$ (in O$_2$) isoflurane and a 6 mm diameter craniotomy was performed with a drill. A coverslip was implanted on the craniotomy region and a head-post was cemented to the skull for head fixation. In-vivo calcium imaging (30 Hz single-plane imaging) was carried out on awake mice without anesthesia 2–3 weeks after the head-post surgery. The imaging of dendritic spines in cortical layer 1 (20–60 mm below the brain surface) required an adequate spatial sampling rate that was achieved by using large zoom factors. Further details of the dataset can be found in^58,59^.

### Dataset 6: simulated dataset based on optical properties of in-vivo structural imaging experiments

Details of the algorithm to simulate dendrites can be found in^55^, and the code is available upon request from the corresponding author of this study. Briefly, each simulated image is $512\times 512$ pixels with each pixel corresponding to $72\times 72$ nm. Each image contained a single dendrite in the optical plane. Pixel intensities varied around $1000\pm 100$ within the dendrite and around $2000\pm 300$ within the spines. Images were scaled between 0 and 2000, and blurred with a Gaussian point spread function (FWHM $=$ 600 nm). Poisson noise was added to the image dataset with an expected spine density of $2.56\upmu {{\mathrm{m}}^{-1}}$. Each potential synapse was labile, and potential synapse states were evolved for 2000 time steps. During each time step, non-potentiated spines were turned on with a probability of $1\times 10^{-3}$, and potentiated spines were turned off with a probability of $4.1\times 10^{-3}$.

## Discussion

Here we present SpineS, an automatic image analysis tool for the longitudinal quantification of dendritic spine features. The proposed tool yields good results in terms of detection and segmentation accuracy, and run times for spine analysis. Results demonstrate that IFI and FWHM can be used interchangeably for individual volume trend assessment in cases for which the spine maintains a regular spherical shape throughout all the time points, or could be used interchangeably when pooled data is required. SpineS provides a means for post-quality assessment which gives users the flexibility to reject or correct individual spine segmentations and neck paths at any time point. This feature provides an important quality control point of the software, as segmentation quality is subject to fluorescence intensity differences between the spine head and neck, which can vary. Overall, the SpineS toolbox improves the speed of image analysis, reducing analysis time from days to hours, while ensuring the quality of the analysis. Another important advantage of SpineS is that it provides more objective quantification of structure than manual methods. Manual FWHM measurements require the user to orient and place a line through the spine head to determine the center of the spine in each image which can introduce experimental bias.

The novel multi-class dendritic feature classification CNN has been trained with more than 25K manual annotations that improved the speed and accuracy of the analysis pipeline compared to our earlier works^45,60^. The feature classifier is fed by the candidate image patches provided by the SURF detector. The detector occasionally fails to detect some of the spines in the FOV. Future work using either a better feature detector or combining multiple feature detectors can improve the accuracy of the analysis.

## Supplementary Information

Supplementary Information.

## Data Availability

Code and all the raw data with annotations will be available upon publication at https://github.com/argunsah/SpineSv2.
