# Supplementary material for: An interactive time series image analysis software for dendritic spines
Source: Sci Rep. 2022 Jul 20;12:12405. doi: 10.1038/s41598-022-16137-y (PMC9300710; doi:10.1038/s41598-022-16137-y)
Supplement: Supplementary file 1 — Supplementary Information. [file 41598_2022_16137_MOESM1_ESM.pdf]

An interactive time series image analysis software for dendritic spines

Ali Özgür Argunşah[1,2,3,+,*], **Ertunç Erdil**[4,+], **Muhammad Usman Ghani**[5], **Yazmín Ramiro-Cortés**[1,6], **Anna F. Hobbiss**[1], **Theofanis Karayannis**[2,3], **Müjdat Çetin**[7], **Inbal Israely**[1,8], **and Devrim Ünay**[9,10,*]

[1]Champalimaud Research, Champalimaud Centre for the Unknown, 1400-038 Lisbon, Portugal.
[2]Laboratory of Neural Circuit Assembly, Brain Research Institute (HiFo), University of Zürich, Zürich, Switzerland.
[3]Neuroscience Center Zurich (ZNZ), UZH/ETH Zürich, Zürich, Switzerland.
[4]Computer Vision Laboratory, ETH Zürich, Zürich, Switzerland.
[5]Department of Electrical and Computer Engineering, Boston University, Boston, MA 02215, USA.
[6]Departamento de Neurodesarrollo y Fisiología, Instituto de Fisiología Celular, Universidad Nacional Autónoma de México, Ciudad de México, C.P. 04510, Mexico.
[7]Department of Electrical and Computer Engineering and Goergen Institute for Data Science, University of Rochester, Rochester, NY 14627, USA.
[8]Department of Pathology and Cell Biology, Columbia University, New York, NY 10032, USA.
[9]Department of Biomedical Engineering, İzmir University of Economics, İzmir, Turkey.
[10]Department of Electrical and Electronics Engineering, İzmir Democracy University, İzmir, Turkey.
[+]Equal Contribution
[*]Correspondence: argunsah@hifo.uzh.ch, unaydevrim@gmail.com

ABSTRACT

Live fluorescence imaging has shown the dynamic nature of dendritic spines, with changes in shape occurring both during development and in response to activity. The structure of a dendritic spine correlates with its functional efficacy. Learning and memory studies have shown that a great deal of the information stored by a neuron is contained in the synapses. High precision tracking of synaptic structures can give hints about the dynamic nature of memory and help us understand how memories evolve both in biological and artificial neural networks. Experiments that aim to investigate the dynamics behind the structural changes of dendritic spines require the collection and analysis of large time-series datasets. In this paper, we present an open-source software called SpineS for the automatic longitudinal structural analysis of dendritic spines with additional features for manual intervention to ensure optimal analysis. We have tested the algorithm on in-vitro, in-vivo, and simulated datasets to demonstrate its performance in a wide range of possible experimental scenarios.

Study	Modality	Dim.	Image Stack Size Resolution Objective	Exp. Cond.	Flourescence Method	Pre-Processing	Detection	Segmentation	Classification	Tracking	Language and Availability
Levet et al., 2020[1]	STED	2D	Super Resolution (Specs not defined)	Undefined	Undefined	Wavelet	Spline	Delaunay Triangulation	-	-	Java Available
Erdil et al., 2019[2]	2P	2D /3D	1024x1024x20/40 (0.198x0.198x0.5) 60X	Org. Slice Cultures	Dendra/AFP GeneGun	Median Filtering	Manual ROI	Act. Con. + Shape Pri.	-	-	Matlab AUR
Smirnov et al., 2018[3]	2P	2D	128x128xZ (0.067x0.067xz) 60X/30X/15X	Org. Slice Cultures	GFP GeneGun	Median Filtering	Thresholding + ML	Otsu	-	-	Matlab Available
Basu et al., 2018[4]	Confocal	3D	1024x1024xZ (.07x.07x.2) 63X	Dis. Cult.	Syn-GFP Transfection GeneGun	Gaussian De-Noising	Manual ROI	Multi Scale Opening	Rule Based	-	Unspecified
Xiao et al., 2018[5]	2P	2D	1024x1024xZ (0.03x0.03x0.16-0.2 63X	Acute Slice	YFP-H Line	Deconv.	CNN	-	-	-	Unspecified
Rada et al., 2018[6]	2P	2D	1024×1024×20/40 (0.198×0.198×0.5) 60X	Org. Slice Cultures	Dendra2C GeneGun	Median Filtering	Dot Enh. + SIFT + SVM	-	-	ICP Based	Matlab AUR
Ghani et al., 2017[7]	2P	2D	1024×1024×20/40 (0.198×0.198×0.5) 60X	Org. Slice Cultures	Dendra2C GeneGun	DNSM / HOG	Manual ROI	-	KDE / SVM / NN	-	Matlab Available
Singh et al., 2017[8]	2P	3D	Multiple Datasets	*In-Vivo*	Thy1-GFP	Gaussian 3D Anisotropic Filter	Hessian	Rule Based	-	-	Matlab Available
Basu et al., 2016[9]	Confocal	2D	Unspecified (.07x.07x.4) 40X	Dis. Cult.	Syn-GFP	Gauss de-noising	Convolutional Kernels	Convolution Kernels	Rule-based	-	C++/QT Available
Wang et al., 2016[10]	Confocal	2D	1024x1024 (.24x.24) 63X	Dis. Cult.	Lopfectamine Transfection	Median Filtering + Hu Moment	-	Ridge Detection	Twin-SVM	-	Matlab Unspecified
He et al., 2012[11]	Confocal	3D	Unspecified Unspecified 40X	Unspecified	-	Regularized morphological filter	-	Nonlinear degeneration equation	Gaussian curvature based	-	Unspecified Unspecified
Erdil et al., 2012[12]	2P	2D	1024x1024x20/40 (.0198x.0198x.5) 60X	Org. Slice Cultures	Dendra/AFP GeneGun	Median Filtering	-	Otsu + Watershed	-	-	Matlab Available
Jungblut et al., 2012[13]	Confocal	2D	512x512x40 (.07x.07x.5) 63X (.11x.11x3) 40X	Org. Slice Cultures	Thy1-GFP	Median Filtering	Augmented Fast Marching	-	-	-	NeuRA C++/QT Available
Son et al., 2011[14]	Confocal	2D	1112x941 (.107x.107) 60X	Dis. Cult.	GFP	Unsharp Mask Filtering	ISODATA Thresholding	Geodesic Active Cont.	Rule-based w/shape crit.	Opt. Flow	Unspecified Unspecified
Choy et al., 2010[15]	Confocal	2D	Unspecified Unspecified 40X	Unspecified	Dil	Median Filtering	Hessian Rule Based	Watershed	-	-	Matlab Available
Zhang et al., 2010[16]	Confocal	3D	512x512x120 (.06x.06x.12) 100X	Org. Slice Cultures	Dil	3D Conv.	Eigen A. Shape M.	Fast Marching	-	-	Matlab Available
Fan et al., 2009[17]	2P	3D	512x512x(15-20) (.06x.06x.5) Unspecified	In-Vivo	Dextran	Deconv.	Morphology	Hessian	-	ICP	Matlab AUR
Janoos et al., 2009[18]	2P	3D	512x512x12 (.07x.07x1) 40X	Acute Cultures	GFP	Deconv. / Gauss. Smooth.	Active Contour	Global Thresholding	-	-	MATLAB / C++ Unspecified
Rodriguez et al., 2008[19]	Confocal	3D	512x512x100 (.05x.05x.1) 100X	Fixed	Lucifer Yellow	Deconv.	Clustering	Local Thresholding	Rule-based	-	Unspecified Unspecified
Zhang et al., 2007[20]	2P	2D	512x512x11 (.082x.082x1) Unspecified	Unspecified	GFP	Gauss. Conv.	LDA	Global Thresholding / Morphology	-	-	Matlab Available
Cheng et al., 2007[21]	2P	2D	512x512x11 (.082x.082x1) 60X	Unspecified	Unspecified	Median Filtering	Morphology Rule-based	Adaptive Thresholding	-	-	Matlab Available
Bai et al., 2007[22]	2P	2D	512x512x16 (.084x.084x1) 60X	Org. Slice Cultures	GFP GeneGun	Blind Deconv. / Median Filtering / Unsharp Masking	Line detection /Morphology	Global Thresholding / Morphology	-	-	Matlab Available
Weaver et al., 2004[23]	Confocal	3D	1024x1024xZ (.098x.098x.081) 100X	Fixed Tissue	Lucifer Yellow	Blind Deconv.	Morphology	Global Thresholding	Rule-based	-	Unspecified
Koh et al., 2002[24]	2P	2D /3D	Unspecified	Org. Slice Cultures	GFP	Median Filt. / iterative reblurr. deconvolution	Morphology Rule-based	Global Thresholding	Rule-based	Reg. Based	Com.

Supplementary Table 1. A non-exhaustive list of dendritic spine analysis literature from 2000 to 2022. Abbreviations: 2D, two-dimensional; 3D, three-dimensional; 2P, two-photon; AUR, available upon request; CNN, convolutional neural network; Dis., dissociated; NA, not available; SIFT, scale-invariant feature transformation; GFP, green-fluorescence protein; ICP, iterative closest point; Com., commercialized; SVM, support vector machines; Org., organotypic; Cult, culture; ML, machine learning.

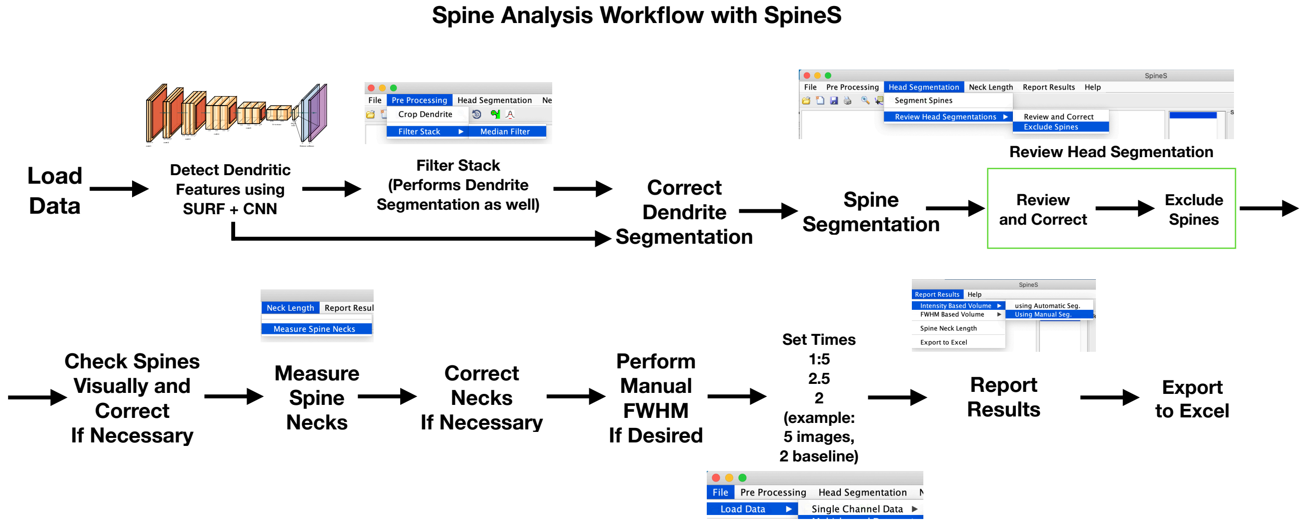

Supplementary Figure 1. Steps to follow to analyze a dataset. The time series z-stack dataset is loaded using the Bio-Formats toolbox. Various dendritic features such as spines, dendrite centers and dendrite edges are detected automatically. Users can correct misdetections of features. Spine head segmentation is performed using detected spine centers via the watershed algorithm. After segmentation, corrections are performed or the user excludes useless spines. Neck-length measurement can now be run. Users can perform FWHM-based volume estimation manually at this point to conclude the analysis before all the quantification is performed and stored in Matlab and Excel formats.

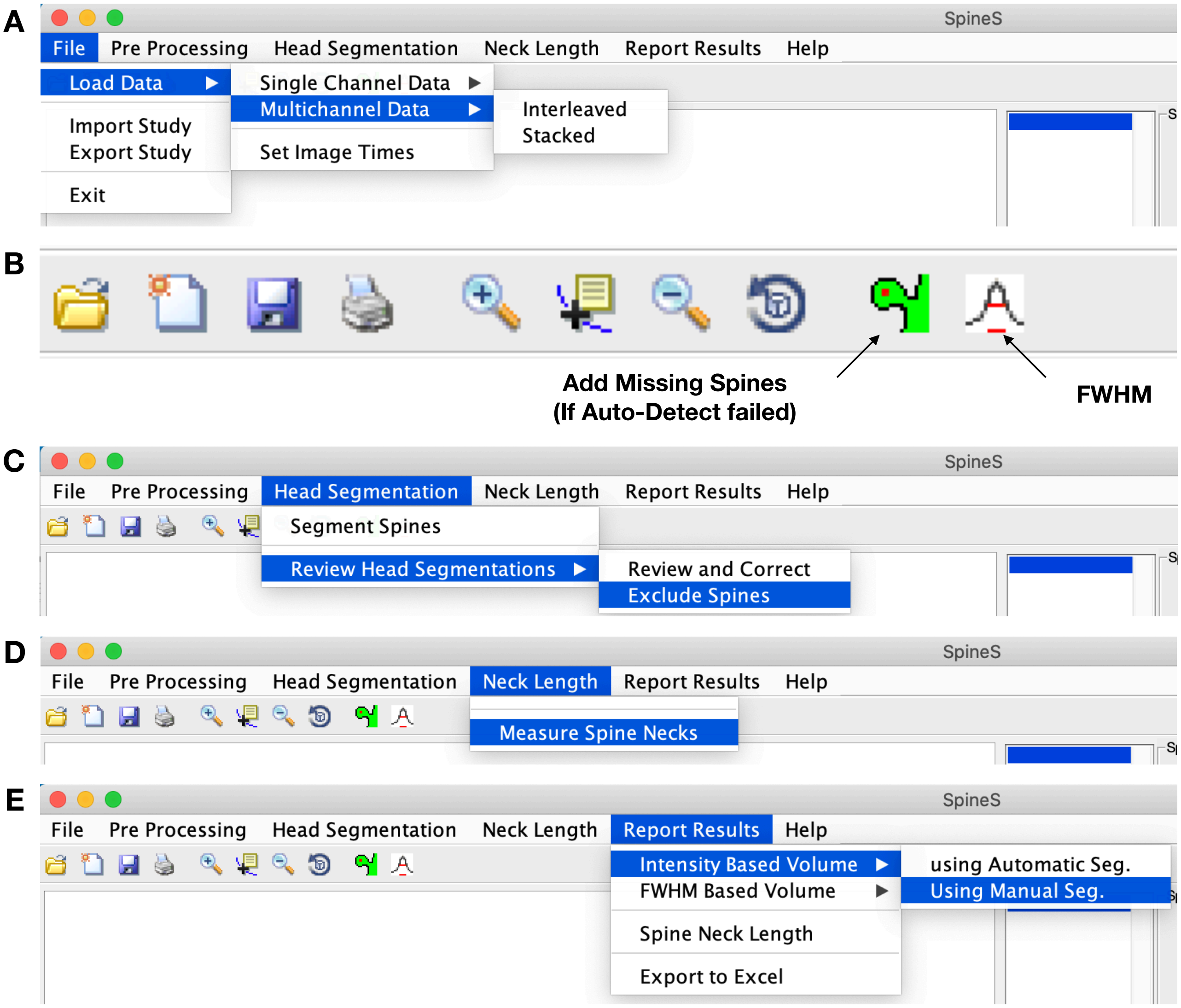

Supplementary Figure 2. SpineS GUI Menu Bar. (A) Single or multichannel times series data can be loaded either using the open microscopy bio-formats plugin or directly from folders containing .tif image stacks. (B) If automatic spine detection fails to detect some spines, the user can add them via *Add Missing Spines* option. Additionally, the toolbar includes spine selection and FWHM buttons in addition to classic Matlab tools. (C) The head segmentation tab includes the *Segment Spines* option to start the segmentation process and global correction as well as *Exclude Spines* from the analysis in case of erroneous spine segmentation. (D) After the dendrite and spine head segmentations are completed and corrections are performed, the *Measure Spine Neck* function can be executed to compute spine neck paths. (E) When all previous steps are completed, the user can plot the results and export them to an Excel format.

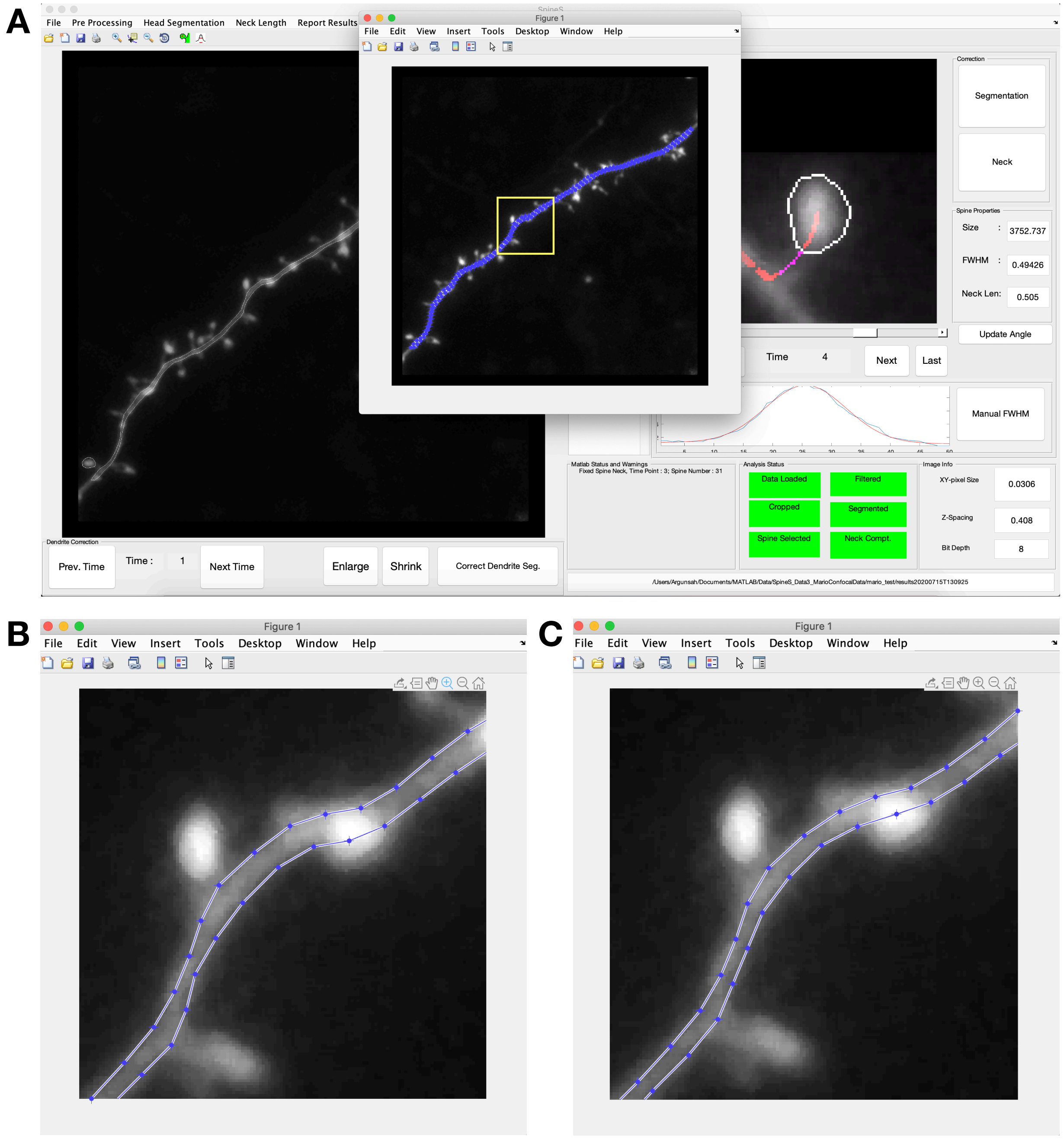

Supplementary Figure 3. Dendrite segmentation correction. (A) The *Correct Dendrite Seg.* button opens a new window with the segmented dendrite in discreet movable points. The user should move imperfect points by holding the left mouse button and dragging. Double clicking at one of the points saves the segmentation. (B) Example of an imperfect segmentation. (C) Segmented dendrite after manual intervention.

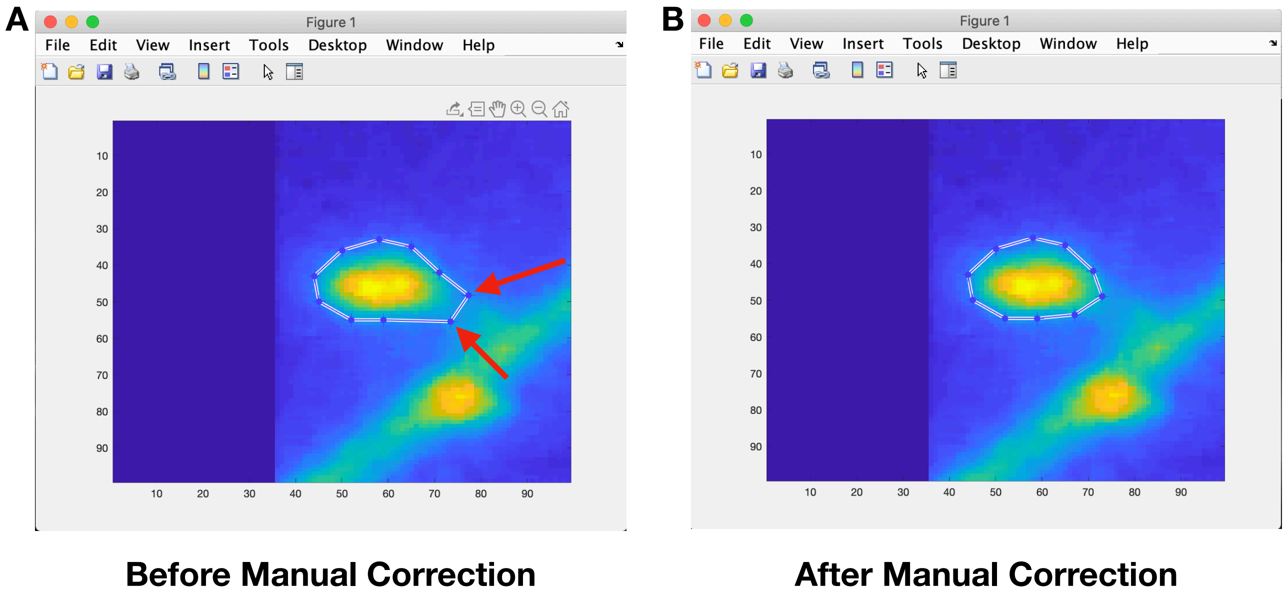

Supplementary Figure 4. Spine segmentation correction. The *Segmentation* button under the Correction panel opens a new window with the segmented spine in discreet movable points. The user should move imperfect points by holding the left mouse button and dragging. Double clicking at one of the points saves the segmentation. (A) Example of an imperfect segmentation. (B) Segmented spine head after manual correction.

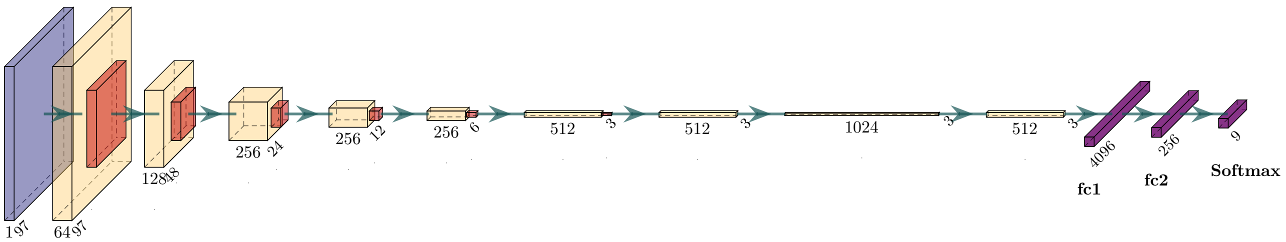

Supplementary Figure 5. Architecture of our dendritic feature detection network. Input images are 97×97 pixel corresponding to $3.395 \times 3.395 \mu m^2$.

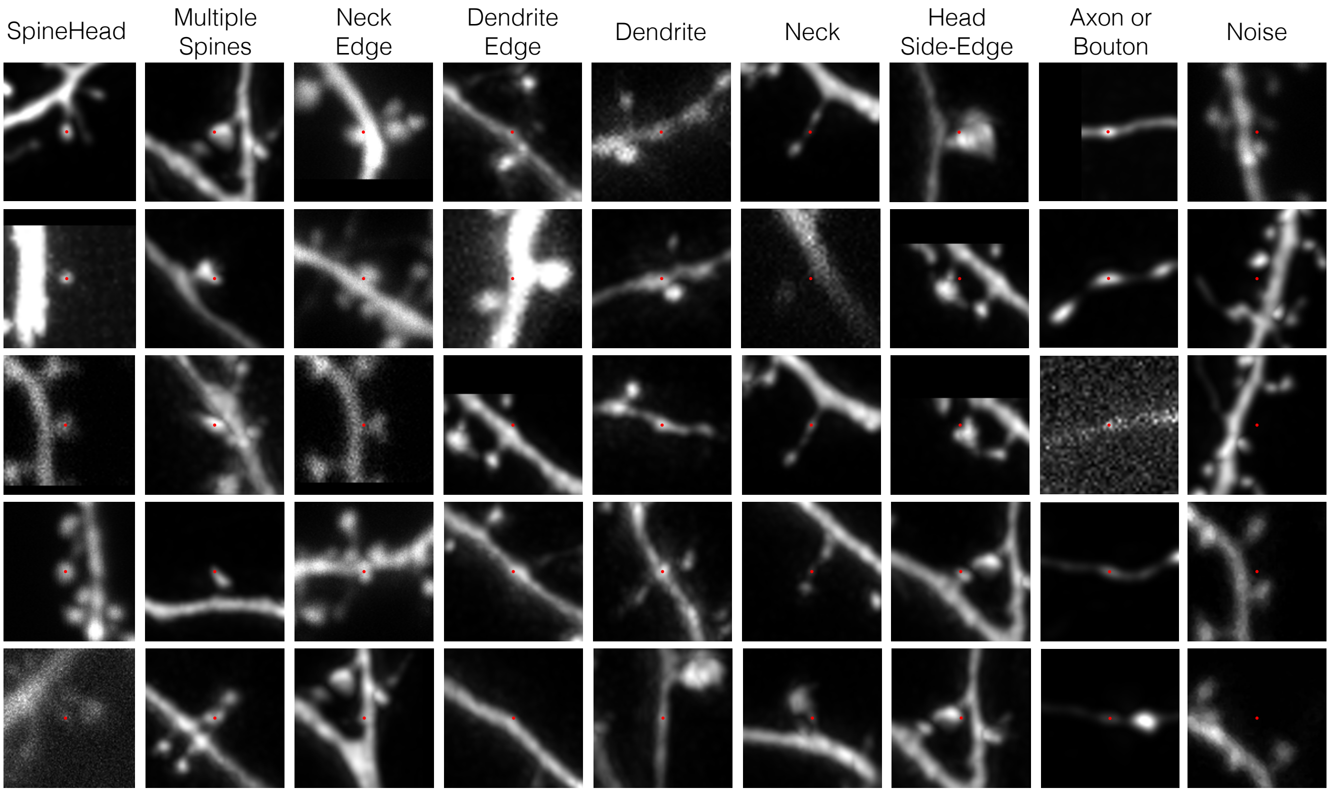

Supplementary Figure 6. Five example images from each of the classes that were used to train the convolutional network. The red dot represents the detected SURF point. ROIs were cropped so that the feature was located in the center. During the CNN training, data augmentation was used such as rotation and flipping but not translation since the center information has to be kept stable. The default MetricThreshold value of the SURF detector has been decreased to 250 from 1000 to increase the number of detected features.

Dendrite	Spine	SpineS vs Manual Intensity	SpineS vs Manual FWHM	Manual Intensity vs Manual FWHM
1	1	97.52	86.34	87.01
	2	88.73	80.23	84.71
	3	91.23	91.12	88.68
	4	94.59	74.72	72.93
2	5	97.09	83.85	84.23
	6	88.70	87.4	90.15
	7	90.81	89.06	88.01
3	8	95.88	90.01	88.31
	9	90.80	82.33	85.52
	10	97.08	79.10	80.44
	11	86.01	90.06	86.27
	12	87.75	78.34	74.54
	13	94.34	78.81	81.16
4	14	88.22	89.12	87.24
	15	83.85	87.35	89.86
5	16	89.37	82.26	82.27
	17	90.33	89.39	87.98
6	18	91.87	87.78	88.19
	19	72.66	69.78	77.61
	20	83.63	84.67	88.86
7	21	94.03	92.66	95.14
	22	78.77	71.41	91.36
8	23	94.09	92.32	93.28
	24	94.56	91.14	90.74
9	25	86.42	84.32	86.00
	26	96.07	67.95	70.77
	27	93.08	84.62	81.52
Mean		90.28	83.93	85.29
S.D.		5.83	6.93	6.01

Supplementary Table 2. Comparison of automatic segmentation with manual segmentation and manual FWHM-based volume estimation methods. Comparisons for all 27 analyzed spines. SpineS: IFI-based volume using automatic segmentations; Manual Intensity: IFI-based volume using manual segmentations by an expert; Manual FWHM: FWHM-based volume quantified by a different expert (Dataset 1).

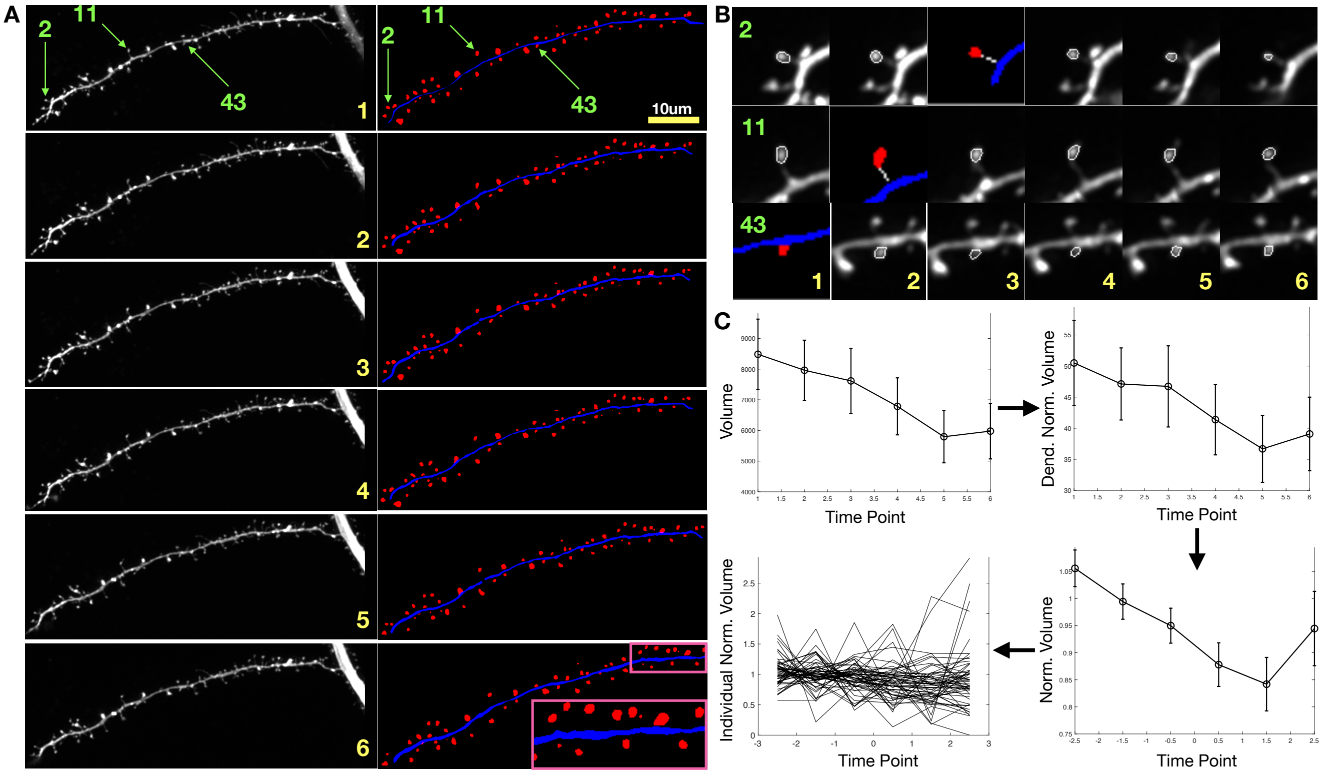

Supplementary Figure 7. Dataset 3 (Chemical LTD) Analysis Results. 57 spines were analyzed at 6 consecutive time points. (A) Images of the dendrite in the left column; segmented dendrite and spines in the right column. Yellow numbers represent time, green numbers represent spines. Spines highlighted in B (2, 11 and 42) are indicated with arrows. (B) Three examples of spines and their segmentations and neck paths. (C) Results of the spine head volumes before and after dendrite normalization (top), and after baseline normalization following dendrite normalization (bottom), on average and individually.

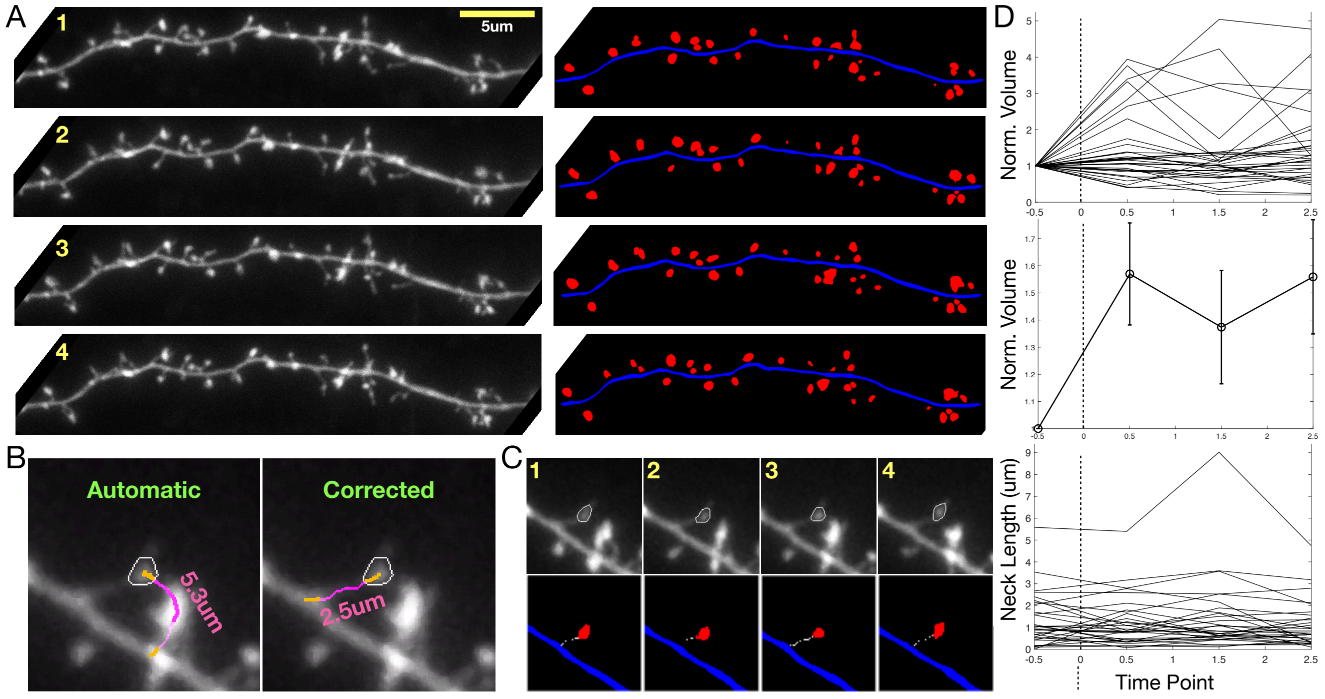

Supplementary Figure 8. Dataset 4 (Chemical LTP) Analysis Results. 36 spines were analyzed at 4 consecutive time points. (A) Images of the dendrite are shown in the left column; segmented dendrite and spines in the right column. Yellow numbers represent time points (B) An example of a bad spine neck path on the left and after manual correction on the right. The spine neck is traced from the center of the spine to the center of the dendrite (orange path) but neck length is computed from the edges of the spine and dendrite segmentations (magenta path). (C) Examples of a segmented spine on top, and segmentation plus neck path on the bottom. (D) Top: Individual spine normalized volume over time, Middle: Average normalized volume over time. Bottom: Individual spine neck lengths.

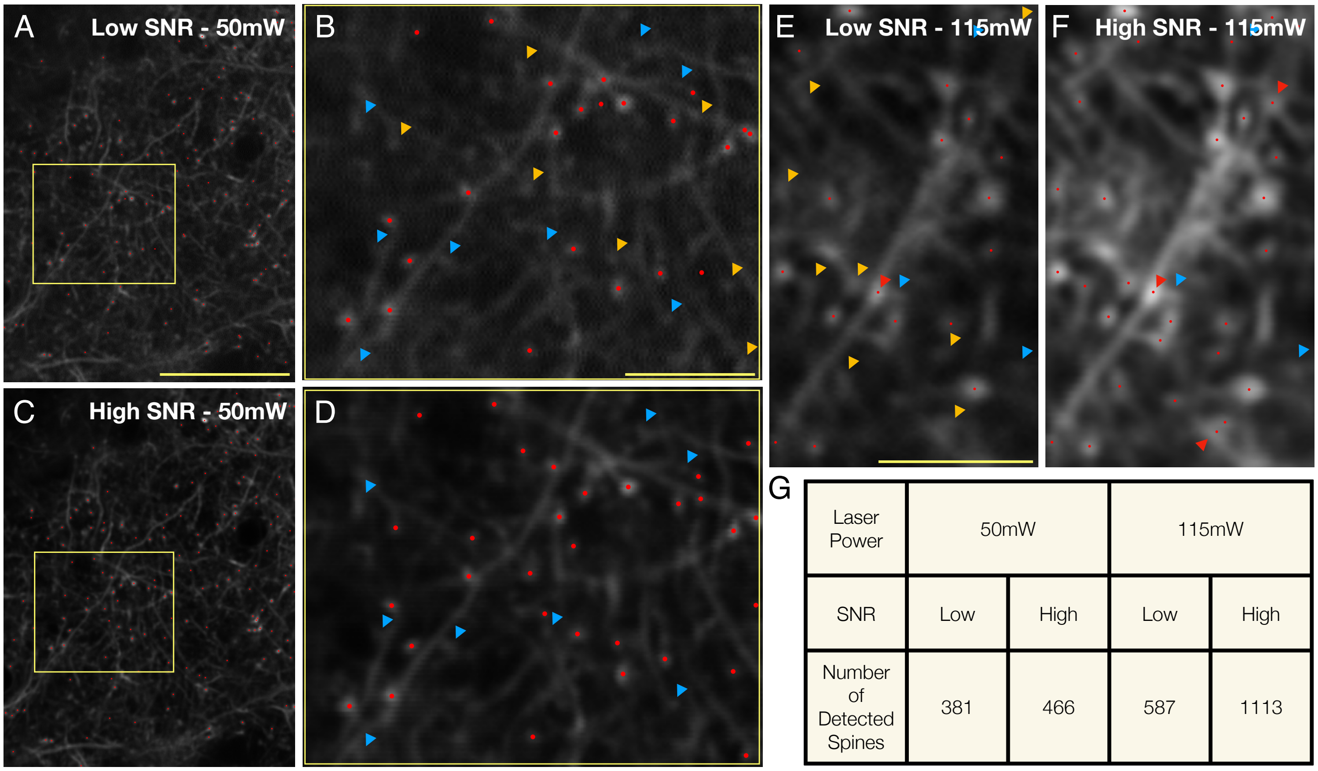

Laser Power	50mW		115mW	
SNR	Low	High	Low	High
Number of Detected Spines	381	466	587	1113

Supplementary Figure 9. In-vivo dendritic calcium imaging dataset[25]. Dataset has four time-series stacks collected using two different imaging laser powers under two different signal-to-noise ratio (SNR) conditions. (A) Low-SNR condition imaged using 50mW laser power. Red dots are detected spines. Scale bar is 20μm. (B) Zoomed version of the portion in A (yellow box). Scale bar is 5μm. (C) High-SNR version (10-fold) of the same field of view (FOV) in A. (D) High-SNR version of the same FOV in D. (E) Low-SNR condition imaged using 115mW laser power. Scale bar is 5μm (F) High-SNR (10-fold) version of the same FOV in E. (G) Detected spines in different imaging conditions. Yellow arrows represent missing spines in low SNR condition only. Blue arrows represent missing spines both in low- and high-SNR conditions. Red arrows represent false positive detections.

References

1. Levet, F., Tønnesen, J., Nägerl, U. V. & Sibarita, J.-B. Spinej: a software tool for quantitative analysis of nanoscale spine morphology. Methods (2020).
2. Erdil, E., Argunsah, A. O., Tasdizen, T., Unay, D. & Cetin, M. Combining nonparametric spatial context priors with nonparametric shape priors for dendritic spine segmentation in 2-photon microscopy images. In 2019 IEEE 16th International Symposium on Biomedical Imaging (ISBI 2019), 204–207 (IEEE, 2019).
3. Smirnov, M. S., Garrett, T. R. & Yasuda, R. An open-source tool for analysis and automatic identification of dendritic spines using machine learning. Plos one **13**, e0199589 (2018).
4. Basu, S. et al. Quantitative 3-d morphometric analysis of individual dendritic spines. Sci. reports **8**, 1–13 (2018).
5. Xiao, X. et al. Automated dendritic spine detection using convolutional neural networks on maximum intensity projected microscopic volumes. J. neuroscience methods **309**, 25–34 (2018).
6. Rada, L. et al. Tracking-assisted detection of dendritic spines in time-lapse microscopic images. Neuroscience **394**, 189–205 (2018).
7. Ghani, M. U. et al. Dendritic spine classification using shape and appearance features based on two-photon microscopy. J. neuroscience methods **279**, 13–21 (2017).
8. Singh, P., Hernandez-Herrera, P., Labate, D. & Papadakis, M. Automated 3-d detection of dendritic spines from in vivo two-photon image stacks. Neuroinformatics **15**, 303–319 (2017).
9. Basu, S. et al. 2dspan: semiautomated 2-d segmentation, classification and analysis of hippocampal dendritic spine plasticity. Bioinformatics **32**, 2490–2498 (2016).
10. Wang, S. et al. Morphological analysis of dendrites and spines by hybridization of ridge detection with twin support vector machine. PeerJ **4**, e2207 (2016).
11. He, T., Xue, Z. & Wong, S. T. A novel approach for three dimensional dendrite spine segmentation and classification. In SPIE Medical Imaging, 831437–831437 (International Society for Optics and Photonics, 2012).
12. Erdil, E. et al. A tool for automatic dendritic spine detection and analysis. part i: Dendritic spine detection using multi-level region-based segmentation. In IEEE Proc. International Conference on Image Processing Theory, Tools and Applications (IPTA) (2012).
13. Jungblut, D. et al. Spinelab: tool for three-dimensional reconstruction of neuronal cell morphology. J. Biomed. Opt. **17**, 0760071–0760077 (2012).
14. Son, J., Song, S., Lee, S., Chang, S. & Kim, M. Morphological change tracking of dendritic spines based on structural features. J. Microsc. **241**, 261–272, DOI: 10.1111/j.1365-2818.2010.03427.x (2011).
15. Choy, S. et al. Multi scale and slice-based approach for automatic spine detection. In Engineering in Medicine and Biology Society (EMBC), 2010 Annual International Conference of the IEEE, 4765 –4768, DOI: 10.1109/IEMBS.2010.5626640 (2010).
16. Zhang, Y. et al. A neurocomputational method for fully automated 3d dendritic spine detection and segmentation of medium-sized spiny neurons. NeuroImage **50**, 1472 – 1484, DOI: 10.1016/j.neuroimage.2010.01.048 (2010).
17. Fan, J., Zhou, X., Dy, J., Zhang, Y. & Wong, S. An automated pipeline for dendrite spine detection and tracking of 3d optical microscopy neuron images of in vivo mouse models. Neuroinformatics **7**, 113–130 (2009).
18. Janoos, F. et al. Robust 3d reconstruction and identification of dendritic spines from optical microscopy imaging. Med. Image Analysis **13**, 167–79 (2009).
19. Rodriguez, A., Ehlenberger, D. B., Dickstein, D. L., Hof, P. R. & Wearne, S. L. Automated three-dimensional detection and shape classification of dendritic spines from fluorescence microscopy images. PLoS ONE **3**, e1997 (2008).
20. Zhang, Y. et al. Dendritic spine detection using curvilinear structure detector and lda classifier. NeuroImage **36**, 346 – 360, DOI: 10.1016/j.neuroimage.2007.02.044 (2007).
21. Cheng, J. et al. A novel computational approach for automatic dendrite spines detection in two-photon laser scan microscopy. J. Neurosci. Methods **165**, 122 – 134, DOI: 10.1016/j.jneumeth.2007.05.020 (2007).
22. Bai, W., Zhou, X., Ji, L., Cheng, J. & Wong, S. T. C. Automatic dendritic spine analysis in two-photon laser scanning microscopy images. Cytom. Part A **71A**, 818–826, DOI: 10.1002/cyto.a.20431 (2007).

23. Weaver, C. M., Hof, P. R., Wearne, S. L. & Lindquist, W. B. Automated algorithms for multiscale morphometry of neuronal dendrites. Neural Comput. **16**, 1353–1383 (2004).

24. Koh, I. Y., Lindquist, W. B., Zito, K., Nimchinsky, E. A. & Svoboda, K. An image analysis algorithm for dendritic spines. Neural computation **14**, 1283–1310 (2002).

25. Xinyang, L. Deepcad-rt dataset: mouse dendritic spines. Zenodo DOI: 10.5281/zenodo.6275571 (2022).
